# Bite of a Copperhead Snake

**Published:** 1853-06

**Authors:** 


*Bite of a Copperhead Snake (Trigonocephalus Contortix) successfully treated with Whisky.*—Dr. N. H. Moragne, of Abbeville, South Carolina, relates (*Southern Med. and Surg. Journal*, Feb. 1853) the following case:

"On the 21st of June last, I was called to see a negro man belonging to Capt. P——, of Abbeville district. Found him partially delirious; skin hot and dry; pulse very much excited, ranging from 100 to 120; left leg and ankle swollen to a great degree. Upon making inquiry into the history of this case, I learned that the patient had been bitten about twelve hours previously by a 'trigonocephalus,' or, as it is frequently styled in this part of the country, copperhead or highland mockeson. This very poisonous reptile was concealed beneath the step of a *meat-house*, and inflicted a wound upon the inside of the foot, near the ankle-joint. I immediately applied a ligature above the seat of affection; prescribed poultices over the wound; and olive oil, ammonia, &c., internally.

"22d. The patient is *in statu quo;* no abatement of the swelling, delirious; ordered whisky, *ad libitum.*

"23d. No decided improvement; still anxious, restless, and uneasy; skin hot and dry. Continued the whisky, combined with capsicum; it was administered until the patient was fully under its influence, without regard to quantity. Left opium to be given if necessary.

"24th. Had passed the "crisis." A profuse perspiration was out over his entire system; the tumefaction was subsiding; the delirium had ceased; he spoke rationally, and speedily convalesced."

